# Relationship between morphometric measurements and blood parameters in horses with varying adiposity levels and physiological conditions

**DOI:** 10.1002/vms3.70024

**Published:** 2024-10-09

**Authors:** Arash Omidi, Aria Rasooli, Saeed Nazifi, Abbas Heydari, Mohammad Seirafinia

**Affiliations:** ^1^ Department of Animal Health Management School of Veterinary Medicine Shiraz University Shiraz Iran; ^2^ Department of Clinical Sciences School of Veterinary Medicine Shiraz University Shiraz Iran; ^3^ School of Veterinary Medicine Shiraz University Shiraz Iran

**Keywords:** adiposity, blood, equine, morphometry, physiology

## Abstract

**Background:**

Understanding and finding the correlation between morphometric measurements and horse blood parameters is crucial for predicting equine metabolic issues.

**Objective:**

This study aims to analyse morphometric measurements and blood samples in horses with varying adiposity levels.

**Study design:**

Cross‐sectional observational.

**Methods:**

A total of 50 horses were included in the study and categorized into groups based on their body condition score (BCS) and cresty neck score (CNS).

**Results:**

The insulin concentration was significantly higher in overweight horses (*p* = 0.022). Female horses exhibited higher cortisol concentrations (*p* = 0.025) and girth circumference at the withers (*p* = 0.004) compared to males. Lactating mares exhibited higher concentrations of serum total protein (*p* = 0.012) and globulin (*p* = 0.003). A positive correlation was observed between BCS and insulin concentrations (*r* = 0.290, *p* = 0.041). Negative correlations were found between neck circumference to height at withers and glucose (*r* = −0.309, *p* = 0.029), CNS and glucose (*r* = −0.315, *p* = 0.026) as well as between crest diameter and cortisol (*r* = −0.360, *p* = 0.01).

**Main limitations:**

Increasing the sample size and conducting longitudinal studies would enhance the study's validity and reliability.

**Conclusion:**

Although insulin, glucose and cortisol concentrations have predictive capabilities based on signs and certain morphometric measurements, their correlations are not always strong. Therefore, this study challenges the notion that all overweight horses are unhealthy, as overweight horses can still have good metabolic health. Conversely, lean horses may also experience metabolic issues. Hence, relying solely on visual cues is insufficient to diagnose the metabolic status of horses. Other factors must also be considered to assess their health status accurately.

## Graphical abstract

Exploring correlations between horse morphometrics and blood parameters in relation to fatness levels revealed elevated insulin in obese horses, higher cortisol and girth circumference in females vs. males and elevated serum total protein and globulin in lactating mares.

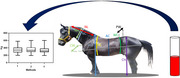

## 1 INTRODUCTION

Obesity in horses is a growing concern due to its association with metabolic and physiological changes akin to those seen in humans (Ribeiro et al., 2021). A horse's health can be affected by conditions like equine metabolic syndrome (EMS), insulin resistance and pituitary pars intermedia dysfunction, which can be associated with various health problems, such as obesity, laminitis and other metabolic and hormonal imbalances (Hart et al., 2016). Research in this field focuses on uncovering the underlying causes and mechanisms of these conditions, aiming to develop effective management and treatment strategies. This involves investigating the effects of interventions like dietary changes, exercise regimens, medications and other strategies on enhancing metabolic health in horses (Johnson et al., 2012). EMS is characterized by various metabolic abnormalities, such as insulin resistance, hyperinsulinemia, abnormal blood lipid concentrations and general or regional adiposity, often manifesting as a cresty neck. The development of this disease can be influenced by various factors, making it challenging to manage effectively. However, with proper management techniques, it is possible to prevent the onset of laminitis in horses affected by EMS and improve their overall health (Stefaniuk‐Szmukier et al., 2023). Equine veterinarians have been interested in discovering simpler methods like the body condition score (BCS) and cresty neck score (CNS) and morphometric measurements to act as early predictors of metabolic issues in horses. A recent study by Daradics et al. found positive associations between non‐esterified fatty acid (NEFA) concentrations and body weight, BCS and CNS. Higher NEFA concentrations were correlated with increased body weight, higher BCS and a more pronounced crest neck (Daradics et al., 2022). Żak et al. divided horses into two groups, EMS and healthy, based on the BCS, CNS and the concentration of insulin. They suggest a strong association between EMS and inflammation. Horses with EMS were found to have significantly higher concentrations of pro‐inflammatory cytokines and acute‐phase proteins than healthy horses (Żak et al., 2020). Previous studies on ponies have shown interesting results, suggesting that insulin dysregulation can be predicted using CNS. Fitzgerald et al. (2019) discovered that certain morphometric factors, including a BCS of 7 or higher, a CNS of 4 or higher and a neck circumference (NC) to height at withers (HW) ratio (NCHW) greater than 0.71, can serve as predictors of clinical laminitis. The debate over using morphometric measurements and blood parameters in horses as indicators of health and performance revolves around their reliability and accuracy. Although some see them as valuable for identifying health issues and predicting performance, others doubt their validity in reflecting true health status or athletic potential. Disagreements also arise over measurement standardization and blood parameter interpretation, leading to inconsistent results and conclusions. Overall, the controversy lies in how these measurements are interpreted and applied in assessing equine health and performance.

This paper aims to investigate the relationship among morphometric measurements, blood parameters and physiological conditions in horses with varying levels of adiposity. By exploring how age, gender and physiological factors impact these variables, the study seeks to identify potential predictors for changes in blood parameters and contribute to the development of improved management strategies for equine obesity and related metabolic disorders.

## 2 MATERIALS AND METHODS

This study evaluated 50 horses from Shiraz horse farms, consisting of 15 Arabian, 15 Dareshouri, 3 Turkmen, 7 Kurdish and 10 non‐native breeds. Different morphometric measurements were carried out utilizing standard techniques (Carter, Geor, et al., 2009; Ghezelsoflou et al., 2018), including HW, defined as the distance between the withers to the ground, body length (L), defined as the distance between the point of the shoulder to the point of the buttock, NC, defined as the circumference of midway point between the poll and the withers, girth circumference (GC), defined as the circumference of the thorax along the point of withers and olecranon tuber, abdomen circumference, defined as at the circumference of widest point of the trunk and read at maximum expiration, croup height, defined as distance from the ground straight up to the highest point of the croup (tuber sacrale of the ilium), waist circumference, defined as around the abdomen coinciding with the point of the umbilicus at two thirds of the distance from the point of the shoulder to the point of the hip (tuber coxae) and at the end of the expiratory pause, neck height (NH), defined as the measurement taken along a line perpendicular to the neck length (NL) at its midpoint, NL, defined as the distance between poll to withers, chest width, defined as the distance between two outer points of the humeral bones from frontview and pelvic width, defined as the distance between the right and left coxal tuber of ilium, were measured by meter. NC to height of neck (NCHN), NCHW and GC to HW ratio (GCHW) were calculated based on the measurements mentioned. The subjects were standing quietly for the measurements.

**FIGURE 1:**
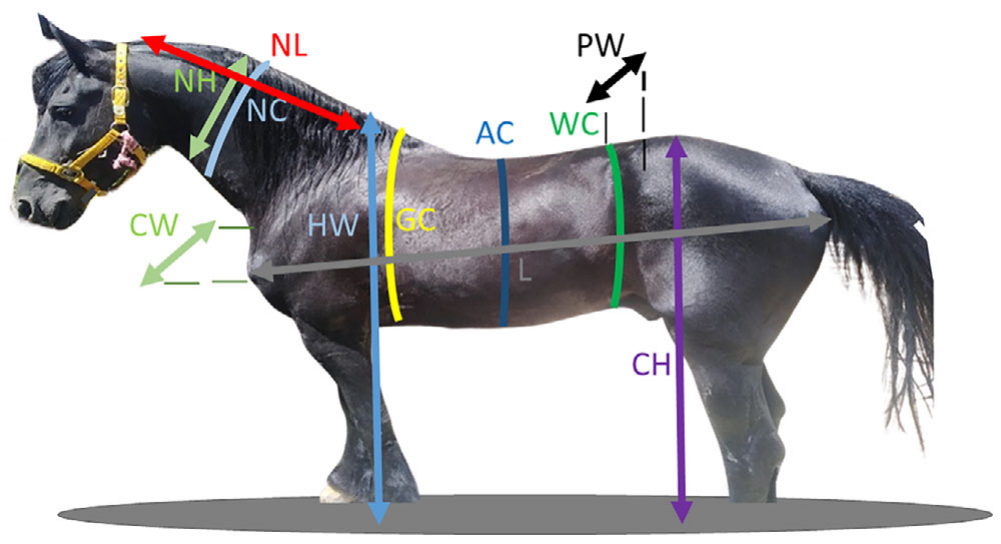
Morphometric measurement sites in the horse standing quietly with the neck positioned at approximately 45°C relative to the horizon. AC, abdomen circumference; CH, croup height; CW, chest width; GC, girth circumference; HW, height at withers; L, body length.; NC, neck circumference; NH, neck height; NL, neck length; PW, pelvic width; WC, waist circumference.

The height measurements were taken using a graduated measuring stick, whereas the length and circumference measurements were done using a flexible tape. Additionally, calibrated callipers were employed for the width measurements. The measurement criteria for various parts of the horse's body are illustrated in Figure 1.

Weight estimation in the study was conducted through three different methods. The first method involved visual estimation, where observers visually assessed and estimated the weight of the horses. This visual estimation was performed by at least three individuals, and the average of their estimations was recorded. The second method utilized a specific meter (weight tapes) to measure the chest circumference of the horses, which was then used to estimate their weight. The third method involved calculating the weight based on a formula that took into account the horse's chest girth and body length. This formula (Wagner & Tyler, 2011), weight (kg) = (heart girth [cm] × body length [cm] × body length [cm])/11,880, was used to estimate the weight when a scale was not available. These methods are commonly employed in routine practice to determine the body weight of horses in situations where a scale is not accessible. Neck diameter (ND) and crest diameter were measured using a calliper. Henneke's BCS and Carter's CNS were evaluated by an expert veterinarian using the 9‐level Henneke system based on visual and tactile assessments. The CNS score ranged from 0 to 5, indicating no fat accumulation in the neck region and severe fat accumulation in the neck region, respectively (Busechian et al., 2022; Carter, Geor, et al., 2009; Carter, Treiber, et al., 2009; Geor et al., 2013; Henneke Potter et al., 1983).

The horses were classified into three groups based on their BCS and CNS, with the first group consisting of 31 horses that had a normal body status, characterized by a BCS lower than 7 and a CNS of 3 or lower. The second group comprised five horses without generalized obesity, possessing a BCS lower than 7 but a CNS greater than 3, indicating regional fat accumulation. The third group, which consisted of 14 horses, was identified to be overweight, as they had a BCS of 7 or higher and a CNS of 1 or higher. Finally, a 10 mL blood sample was collected between 0800 and 1000 from the jugular vein of relaxed horses. The horses were kept in stalls overnight and had free grass hay and water access, but no concentrated feed was provided. All horses were in good health, and no lameness was observed. Samples were analysed in the laboratory after the serum had been separated and stored at −21°C until the serum insulin, glucose, cortisol, triglycerides, cholesterol, total protein and albumin were measured. The following blood parameters were measured using these methods: Serum cortisol was measured using a solid‐phase sandwich ELISA method using a commercial ELISA kit (Monobind Inc., Catalogue Number: 3625‐300A). The sensitivity of the kit was 0.25 µg/dL. Insulin was measured using a horse solid‐phase sandwich ELISA method (Shanghai Crystal Day Biotech Company): insulin intra‐assay: CV < 8%; inter‐assay: CV < 10%; insulin sensitivity: 3 µU/mL. Serum total cholesterol, triglycerides, glucose, total protein and albumin were measured with commercial kits (Pars Azmoon Co.) and were analysed using a biochemical Autoanalyzer (Alpha Classic AT, Sanjesh Company). The difference between total protein and albumin estimated serum globulin concentration.

### 2.1 Statistical analysis

In this study, horses were classified based on various criteria, including natural horses, regional obesity horses and obese horses. Gender, age and physiological conditions were also considered. The data were analysed using SPSS software (version 22.0; IBM Corp.), and the Shapiro–Wilk test was used to evaluate the normal distribution. Mean and standard deviation were calculated for normally distributed data, whereas median and range were calculated for non‐normally distributed data. Logarithmic values were used to achieve normal distribution if needed. Despite the logarithmic transformation, the CNS variable was not normally distributed and was analysed using non‐parametric methods. Independent *t* test was used to compare two groups based on gender and age. A one‐way ANOVA test was used to compare means in multiple groups considering different physical and physiological conditions. The post hoc Tukey test was then used to identify significant differences among specific groups. The Mann–Whitney *U* and Kruskal–Wallis tests were used for non‐parametric data. A value of *p* ≤0.05 was considered statistically significant.

## 3 RESULTS

In the study, 50 equines were evaluated, with 32 (64%) female and 18 (36%) male. The animals were in different age groups, with 14 individuals (28%) being between 1 and 3 years, 16 individuals (32%) being between 3 and 6 years and 20 equines (40%) being between 7 and 24 years. Horses up to 4 years old were classified as young (*n* = 24; mean ± SD = 32.88 ± 15.93; median = 36; range = 46 months), whereas those above 4 years old were classified as adults (*n* = 26; mean ± SD = 126 ± 64.06; median = 108; range = 228 months). Table 1 presents the distribution of animals among different body condition categories. All observed horses had a BCS higher than 4 (mean ± SD = 6.2 ± 0.75; median = 6. range = 3). Thirty‐nine horses had a CNS score of 3 or lower, with a mean of 2.81 ± 0.27, a median of 3 and a range of 1, whereas 11 horses had a CNS score of 3.5.

**TABLE 1:** Distribution of body condition scores (BCS) in some horses in the city of Shiraz, Iran.

Body condition	BCS	Total	
		*N*	%
**Thin**	<4	0	0
**Great**	4.5–6	28	56
**Overweight**	6.5–7	19	38
**Obese**	7.5–9	3	6

The morphometric assessments indicated that overweight horses typically exhibited elevated values for most of the measurements, a result that aligns with common sense expectations. Notably, the NCHN, NCHW and ND consistently displayed uniform values across all three groups. Table 2 indicates that insulin concentrations were considerably greater in overweight horses in comparison to the other two groups (*p* = 0.022). Additionally, although cortisol concentrations seemed to be numerically higher in the overweight horses, there was no statistically significant difference. The remaining biochemical parameters listed in Table 2 did not exhibit significant differences.

**TABLE 2:** Comparison of biochemical parameters in horses among different body conditions.

Parameter (unit)	Normal (*n* = 31)	Regional fat accumulation (*n* = 5)	Overweight (*n* = 14)	*p* value
**CORT (µg/dL)**	15.71 ± 12.47*	12.31 ± 3.34	17.27 ± 13.58	NS
**INS (mU/L)**	3.61 ± 0.84^a^	3.02 ± 1.14^a^	5 ± 2.87^b^	0.022
**GLU (mg/dL)**	84.82 ± 34.18	61.40 ± 6.74	75.07 ± 22.21	NS
**TG (mg/dL)**	21.72 ± 11.38	23.08 ± 11.18	27.56 ± 23.74	NS
**CHOL (mg/dL)**	71.23 ± 31.21	84.80 ± 36.16	72.39 ± 42.82	NS
**TP (g/dL)**	6.04 ± 2.59	6.26 ± 2.42	4.92 ± 1.88	NS
**ALB (g/dL)**	3.32 ± 1.26	3.70 ± 1.35	2.92 ± 1.28	NS
**GLB (g/dL)**	3.73 ± 1.42	2.56 ± 1.07	1.99 ± 0.74	NS

*Note*: Normal: BCS < 7, CNS ≤ 3; regional fat accumulation: BCS < 7, CNS > 3; overweight: BCS ≥ 7, CNS ≥ 1. Different letters in each row indicate statistically significant differences (*p* ≤ 0.05).

Abbreviations: ALB, albumin; CHOL, cholesterol; CORT, cortisol; GLB, globulin; GLU, glucose; INS, insulin; NS, not significant; TG, triglyceride; TP, total protein.

* Mean ± SD.

The study's results suggest that, when comparing male and female horses, only cortisol and GCHW were statistically significant (*p*< 0.05). Specifically, the mean cortisol concentration was higher in females (18.66 ± 14.12 µg/dL) than in males (10.73 ± 4.17 µg/dL), indicating that females had higher cortisol concentrations compared to males. Additionally, the female participants demonstrated a slightly higher GCHW (median 1.13) than the male participants (median 1.09). It is important to note that the remaining parameters did not demonstrate statistically significant differences between male and female horses (Table 3).

**TABLE 3:** Comparison of biochemical parameters in female and male horses.

Parameter (unit)	Female (*n* = 32)	Male (*n* = 18)	*p* value
**CORT (µg/dL)** *	18.66 ± 14.12	10.73 ± 4.17	0.025
**INS (mU/L)**	3.95 ± 2.04	3.92 ± 1.29	NS
**GLU (mg/dL)**	80.16 ± 30.44	79.02 ± 30.38	NS
**TG (mg/dL)**	23.71 ± 18.03	23.09 ± 10.73	NS
**CHOL (mg/dL)**	71.53 ± 37.63	75.36 ± 29.73	NS
**TP (g/dL)**	5.64 ± 2.57	5.95 ± 2.16	NS
**ALB (g/dL)**	3.16 ± 1.29	3.40 ± 1.24	NS
**GLB (g/dL)**	2.48 ± 1.40	2.55 ± 1.01	NS

Abbreviations: ALB, albumin; CHOL, cholesterol; CORT, cortisol; GLB, globulins; GLU, glucose; INS, insulin; NS, not significant; TG, triglycerides; TP, total protein.

* Mean ± SD.

Although significant differences were observed in most morphometric measurements between adult and young horses, parameters, such as BCS, CNS, NCHN, NCHW, crest diameter and neck diameter, did not show significant changes with the growth of the animals. The results indicate no significant differences between adult and young horses were observed in serum biochemical parameters, including cortisol, insulin, glucose, triglycerides, cholesterol, total protein, albumin and globulins. The mean values (median) of morphometric parameters in different physiological states are presented in Table 4. The *p* values for all parameters, except for CNS, NCHN, NCHW, ND and crest diameter, are statistically significant (*p* < 0.05). This indicates significant differences in these morphometric parameters between the different physiological states of the animals. It appears that horses with larger body sizes are more worked compared to other groups, and they engage in more exercise and activity.

**TABLE 4:** Comparison of morphometric parameters in horses across different workloads and lactation.

Parameter (unit)	Maintenance (*n* = 16)	Light work (*n* = 12)	Moderate work (*n* = 14)	In lactation (*n* = 8)	*p* Value
**BCS**	6.00 ± 0.61^*^ (6)^a^	6.17 ± 0.62 (6)^a^	6.86 ± 0.74 (7)^b^	6.06 ± 0.78 (6)^a^	0.017
**CNS**	2.91 ± 0.38 (3)	2.88 ± 0.38 (3)	3.11 ± 0.29 (3)	2.88 ± 0.44 (2.75)	NS
**HW (cm)**	141.83 ± 4.37 (143)^a^	143.00 ± 7.02 (146)^a^	163.79 ± 7.45 (163)^b^	142.26 ± 2.02 (143)^a^	<0.001
NC **(cm)**	83.75 ± 6.99 (84)^a^	85.17 ± 10.12 (88)^a^	95.50 ± 8.23 (95)^b^	80.63 ± 6.63 (82)^a^	0.001
**GC (cm)**	157.44 ± 7.63 (159)^a^	153.83 ± 14.05 (157)^a^	186.36 ± 9.95 (190)^b^	160.88 ± 9.19 (163)^a^	<0.001
**NCHN**	2.31 ± 0.16 (2.32)	2.35 ± 0.12 (2.37)	2.28 ± 0.08 (2.26)	2.28 ± 0.05 (2.29)	NS
**NCHW**	0.59 ± 0.04 (0.59)	0.59 ± 0.05 (0.60)	0.58 ± 0.05 (0.57)	0.57 ± 0.04 (0.58)	NS
**GCHW**	1.11 ± 0.04 (1.11)^ab^	1.07 ± 0.05 (1.07)^b^	1.14 ± 0.04 (1.15)^a^	1.13 ± 0.05 (1.13)^a^	0.024
**Crest diameter (mm)**	6.73 ± 0.98 (6.79)	6.84 ± 0.87 (6.95)	7.22 ± 0.73 (7.18)	6.48 ± 0.50 (6.57)	NS
**Length (cm)**	140.84 ± 7.08 (143)^a^	137.42 ± 5.45 (139)^a^	162.75 ± 9.72 (165)^b^	142.75 ± 4.37 (144)^a^	<0.001
**Croup height (cm)**	140.39 ± 5.69 (141)^a^	145.58 ± 5.42 (146)^a^	162.57 ± 6.32 (160)^b^	140.88 ± 4.52 (142)^a^	<0.001
**AC (cm)**	177.31 ± 7.41 (178)^a^	171.58 ± 12.94 (175)^a^	202.64 ± 12.98 (206)^b^	191.13 ± 9.45 (194)^a^	<0.001
**WC (cm)**	154.53 ± 6.88 (156)^a^	153.00 ± 11.59 (156)^a^	184.07 ± 12.86 (187)^b^	160.25 ± 9.98 (164)^a^	<0.001
**Neck height (cm)**	36.34 ± 3.37 (36.5)^a^	36.17 ± 4.02 (37)^a^	41.86 ± 3.18 (42)^b^	35.25 ± 2.60 (36)^a^	<0.001
**NL (cm)**	42.63 ± 2.25 (43)^a^	43.08 ± 3.50 (43)^a^	51.50 ± 3.67 (52)^b^	40.88 ± 2.90 (41)^a^	<0.001
**CW (cm)**	34.75 ± 3.71 (35)^a^	33.50 ± 3.06 (35)^a^	41.64 ± 2.90 (43)^b^	35.63 ± 2.62 (36)^a^	<0.001
**PW (cm)**	47.13 ± 3.05 (48)^a^	48.33 ± 4.68 (49)^a^	57.79 ± 2.78 (58)^b^	50.00 ± 1.60 (50)^a^	<0.001
**ND (cm)**	18.38 ± 4.16 (17)	17.42 ± 2.64 (18)	19.93 ± 1.44 (20)	17.88 ± 4.67 (16)	NS

*Note*: Different letters in each row indicate statistically significant differences (*p* ≤ 0.05).

Abbreviations: AC, abdomen circumference; BCS, body condition score; CNS, cresty neck score; CW, chest width; GC, girth circumference; GCHW, girth circumference to height at withers; HW, height at the withers; NC, neck circumference; NCHN, neck circumference to height of neck; NCHW, neck circumference to height at withers; ND, neck diameter; NL, neck length; NS, not significant; PW, pelvic width; WC, waist circumference.

* Mean ± SD (median).

The mean values of serum biochemical parameters in different physiological states are presented in Table 5. Significant differences were observed for total protein and globulin between different physiological states. Horses that were in lactation had the highest levels of TP and GLB (*p* values were 0.012 and 0.003, respectively).

**TABLE 5:** Comparative analysis of biochemical parameters in equine under various physiological conditions.

Parameter (unit)	Maintenance (*n* = 16)	Light work (*n* = 12)	Moderate work (*n* = 14)	In lactation (*n* = 8)	*p* Value
**CORT (µg/dL)**	16.83 ± 11.63^*^	10.92 ± 4.31	17.35 ± 13.68	18.37 ± 17.48	NS
**INS (mU/L)**	3.43 ± 0.83	4.02 ± 0.79	4.55 ± 3.07	3.76 ± 1.04	NS
**GLU (mg/dL)**	76.69 ± 24.52	72.33 ± 27.74	79.39 ± 23.15	97.63 ± 48.72	NS
**TG (mg/dL)**	25.33 ± 12.90	20.85 ± 10.92	21.94 ± 22.11	26.50 ± 15.28	NS
**CHOL (mg/dL)**	76.29 ± 35.33	66.21 ± 24.90	65.29 ± 40.31	89.56 ± 35.19	NS
**TP (g/dL)**	6.10 ± 2.36^ab^	4.99 ± 1.69^a^	4.77 ± 2.14^a^	7.91 ± 2.69^b^	0.012
**ALB (g/dL)**	3.53 ± 1.30	2.84 ± 1.06	2.80 ± 1.29	4.06 ± 1.08	0.064
**GLB (g/dL)**	2.57 ± 1.15^a^	2.15 ± 0.76^a^	1.97 ± 0.93^a^	3.85 ± 1.71^b^	0.003

*Note*: Different letters in each row indicate statistically significant differences (*p* ≤ 0.05).

Abbreviations: ALB, albumin; CHOL, cholesterol; CORT, cortisol; GLB, globulin; GLU, glucose; INS, insulin; NS, not significant; TG, triglycerides; TP, total protein.

* Mean ± SD.

The study found that both total protein concentrations and globulin concentrations were significantly higher in the lactation and maintenance conditions compared to the light work and moderate work conditions. CNS, NCHN, NCHW and crest diameter were not affected by gender, age or physiological status. BCS varied across physiological states but was not affected by age and gender. ND changed with varying physiological conditions and age but was not affected by gender. In addition, the study found correlations between various morphometric and biochemical parameters. Positive Spearman's correlation was found between BCS and insulin concentrations (*r* = 0.290, *p* = 0.041). Negative Spearman's correlations were found between NCHW and glucose (*r* = −0.309, *p* = 0.029) and CNS and glucose (*r* = −0.315, *p* = 0.026), as well as between crest diameter and cortisol (*r* = −0.360, *p* = 0.01).

**FIGURE 2:**
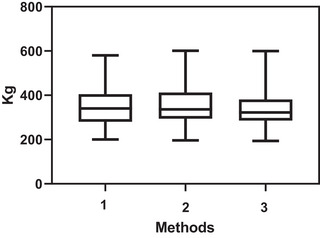
The box plots show no significant difference in weight estimations obtained by the 3 methods used to measure the weight of 50 horses. The methods used were visual inspection (Method 1), weight tape (Method 2) and a regular meter with a formula calculation (Method 3).

A significant correlation was observed between BCS and various weight estimation methods with correlation coefficients ranging from 0.58 to 0.601, and a significant correlation between CNS and various weight estimation methods with correlation coefficients ranging from 0.425 to 0.450 (*p* ≤ 0.001). The Pearson correlation coefficients between three weight estimation methods were studied. The mean values of these variables were 354.10 ± 98.83 kg (Min = 200, *Q*1 = 280, median = 340, *Q*3 = 400 and Max = 580), 356.46 ± 100.19 kg (Min = 196, *Q*1 = 297, median = 336, *Q*3 = 411 and Max = 601) and 345.26 ± 101.03 kg (Min = 193.6, *Q*1 = 288.9, Median = 322.25, *Q*3 = 380.7 and Max = 599.4), respectively. Significant positive correlations were found between these variables, with Pearson correlation coefficients ranging from 0.983 to 0.990. These results suggest a strong association between changes in one variable and the other. Figure 2 shows no significant difference in the weight estimations obtained by the three methods.

## 4 DISCUSSION

Previous research conducted by the authors on horse stables in the Shiraz region revealed a low occurrence of severe weight conditions and cresty necks. This was confirmed through the random selection of horses. The median BCS was recorded as 6, and all CNS were below 3.5. The study found that the overweight group of horses had higher values for most of the morphometric measurements analysed, which is what was expected given their larger body size. The measurements NCHN and NCHW were consistent across all horse groups, and changes in NH and overall horse height from the withers were related to changes in NC. However, any inappropriate changes to the NC can disrupt this relationship. Bruynsteen et al. (2015) found that the weight and morphometric profile of obese Shetland ponies were interrelated. They also found that reducing energy intake could lead to alterations in both BCS and morphometric profiles. The role of adipose tissue in regulating energy balance and insulin secretion through the release of adipokines has been studied in humans and horses. In a group of ponies, some researchers found that clinical laminitis could be predicted accurately by assessing their BCS (≥7), CNS (≥4), and NCHW (>0.71) scores. The CNS was identified as an independent predictor of insulin dysregulation in ponies, according to Fitzgerald et al. (2019). Our study on horses showed that 28% had a BCS of ≥7, none had a CNS score of ≥4 and just 2% had an NCHW score of >0.71. It is worth noting that differences exist in how fat is distributed across the bodies of horses and ponies, with ponies having a higher tendency to gain weight in the neck region due to their smaller size. This may indicate that ponies have a distinct pattern of fat distribution compared to horses, as Frank et al. (2010) suggested. In this study, hyperglycaemia was deemed present if the glucose concentration exceeded 124 mg/dL. Of the horses examined in this study, 8% were found to have hyperglycaemia. It was observed that overweight horses exhibited significantly higher insulin concentrations and tended to have higher cortisol levels than the other groups. The biochemical parameters analysed in horses with varying BCS and CNS showed no significant differences. It is currently defined that fasting hyperinsulinaemia in horses is evident when the fasting insulin concentration is greater than 20 mU/L (Bertin et al., 2018). However, none of the horses in this study met this criterion. The highest insulin concentration observed in this study was 13.90 mU/L. Perhaps one of the contributing factors to the higher insulin concentrations observed in overweight or obese horses is the increased demand for insulin to facilitate glucose absorption in adipose cells (Harris et al., 2020, Ribeiro et al., 2021). Hart et al. (2016) reported similar findings for cortisol concentrations, observing that free cortisol concentrations were higher in overweight/obese healthy horses than in their lean counterparts. The study revealed significant differences between adult and young horses in most morphometric parameters. However, no significant differences were observed in BCS, CNS, NCHN, NCHW, crest diameter and ND, as well as in any of the serum biochemical parameters between adult and young horses. This suggests that although age can impact morphological characteristics in horses, it does not significantly affect the other measurements examined in this study. Jensen et al. (2016) also highlighted the importance of monitoring horses, body condition and morphometric parameters. Their research focused on mature Icelandic horses in Denmark and aimed to evaluate BCS, morphometric measurements and body weight estimation. They found a positive correlation between BCS and certain morphometric measurements, such as GC, NC and body length. It is worth noting that morphometric measurements tend to increase with the size of larger animals and vice versa. However, some measurements expressed as ratios or coefficients, such as NCHN and NCHW, may not change with increased animal size and between adult and young horses. The present study investigated male and female horses and concluded that only cortisol concentrations and the GCHW ratio showed statistical significance. Female horses had higher average cortisol concentrations and a slightly higher GCHW ratio than males. Other parameters analysed showed no significant differences between male and female horses. This topic heavily relies on specific studies conducted on male and female horses, with varying results reported in different studies. Some research has suggested that females’ cortisol concentrations are higher, whereas others have found no significant difference between genders (Aurich et al., 2015; Lelláková et al., 2022). Hormonal differences between male and female horses may affect cortisol secretion, with oestrogen shown to increase cortisol production in some species (Edwards & Mills, 2008). This could potentially explain why female horses have higher cortisol concentrations. Moreover, some studies have suggested that females may be more sensitive to stressful situations, leading to higher cortisol concentrations (Sandanger et al., 2004). The reason for the slightly higher GCHW in female horses than males is unclear and has not been definitively established. However, it may be related to differences in body shape and fat distribution between male and female horses. Jensen et al. (2016) found that the GCHW was a good indicator of obesity in Icelandic horses, with a ratio greater than 1.3 indicating overweight or obese horses. In the present study, the female participants demonstrated a mean GCHW of 1.13 compared to the male participants’ mean of 1.09. The present study revealed that physiological state could significantly impact morphological characteristics in horses, with most morphometric parameters showing significant differences among different physiological states. There were significant variations in total protein and globulin concentrations. These results suggest that the increased demand for protein to support milk production and potential immune response could contribute to the higher total protein and globulin concentrations observed in lactating mares. Therefore, it is important to consider the physiological state of horses when evaluating their morphological characteristics. In a study by Hagawane et al. (2009), serum total protein values were slightly elevated during the early stage of lactation in buffaloes. Similarly, Bobbo et al. (2017) reported that total protein concentrations peaked in dairy cattle's fourth month of lactation. Additionally, the concentration of globulins decreased linearly across lactation, whereas albumin increased with days in milk in a quadratic pattern. These findings underscore the need to account for the physiological state and stage of lactation when interpreting serum protein concentrations in livestock animals. In this study, we observed that the relationship among insulin, glucose and cortisol concentrations and certain signs or morphometric measurements may not always be strong. Specifically, we found that insulin concentrations were significantly higher in overweight horses, indicating a positive correlation between BCS and insulin concentrations (*r* = 0.290, *p* = 0.041). Conversely, negative correlations were found between BCS and glucose concentrations (*r* = −0.315, *p* = 0.026). Multiple factors, including individual variations, environmental factors and other physiological factors, can influence the relationship among these biomarkers. Various studies have supported the notion that insulin dysregulation in overweight or obese horses is influenced by multiple factors. For example, Pardié et al. (2023) demonstrated that pregnant mares with higher BCS had elevated glucose and insulin concentrations compared to mares with normal BCS. Ribeiro et al. (2021) found that horses exposed to a hypercaloric diet exhibited insulin dysregulation, characterized by increased basal glucose and insulin concentrations. Furthermore, Kaufman et al. (2023) observed that horses with a history of laminitis and greater adiposity had higher insulin concentrations in response to grazing forage. Breuhaus (2019) reported that Paso Fino horses exhibited insulin dysregulation compared to Thoroughbreds, with overweight Paso Finos showing even greater insulin dysregulation. Similarly, Bamford et al. (2015) found that increased adiposity did not necessarily lead to reduced insulin sensitivity in obese horses, suggesting that obesity alone may not be solely responsible for lower insulin sensitivity. The observed weak correlation between insulin concentrations, glucose and morphometric variables can be attributed to various factors. Although increased insulin demand in fat cells may contribute to insulin dysregulation, it is crucial to consider other factors such as breed, diet and adiposity. These factors play significant roles in the metabolic imbalance observed in overweight or obese horses. By taking these factors into account, we can gain a more comprehensive understanding of the complex relationship among insulin, glucose and morphometric variables in horses.

The study concluded that all three weight estimation methods in horses are reliable and consistent, as positive correlations were found among them. The study also found that these methods can accurately estimate weight differences among different groups and between adult and young horses, providing valuable information. When executed by expert professionals, the study established the reliability of visual estimation, revealing it to be a simple and expeditious method that does not necessitate specialized equipment. As a result, expert professionals should perform visual estimation to ensure accuracy, despite the previous belief that it needed to be more reliable. These findings have significant implications for equine professionals who must estimate horse weights accurately for various purposes, such as medication dosing or feed requirements. Any of these weight estimation methods can be chosen by equine professionals based on their convenience and availability, given that most equestrians need access to suitable weighing scales for horses or they are unavailable in field conditions.

The current research had limitations due to the small number of horses available. There also needed to be more weighing of the horses using a scale. In addition, the random selection of horses in the stables did not include many very thin or overweight horses.

In conclusion, the findings of the study indicate that overweight horses exhibited elevated insulin concentrations. However, it was observed that certain horses had high BCS but not high CNS, and only a small percentage had high NCHW. Positive correlations were found between BCS and CNS when using weight estimation methods. Conversely, negative correlations were observed between NCHW and glucose, CNS and glucose, crest diameter, and cortisol. The study challenges the assumption that all overweight horses are unhealthy, as some overweight horses can still have good metabolic health. On the other hand, lean horses may also experience metabolic issues. Hence, relying solely on visual cues is insufficient to accurately diagnose the metabolic status of horses. To assess their health status accurately, other factors must be considered in addition to visual observations, signs and specific morphometric measurements. It is recommended that future studies employ larger sample sizes and encompass diverse horse breeds. Moreover, enhancing precision in measurements through the utilization of three‐dimensional imaging tools is proposed to facilitate a more thorough and precise investigation of the correlation between morphological assessments and blood parameters in horses with varying levels of adiposity.

## AUTHOR CONTRIBUTIONS

**Arash Omidi**: Conceptualization; data curation; formal analysis; funding acquisition; investigation; methodology; project administration; resources; software; supervision; validation; visualization; writing–original draft; writing–review and editing. **Aria Rasooli**: Formal analysis; funding acquisition; investigation; project administration; supervision; writing–review and editing. **Saeed Nazifi**: Data curation; formal analysis; methodology; validation; visualization; writing–review and editing. **Abbas Heydari**: Data curation; investigation; methodology; writing–review and editing. **Mohammad Seirafinia**: Investigation; methodology; validation; visualization; writing–review and editing.

## CONFLICT OF INTEREST STATEMENT

The authors declare no conflicts of interest.

### ETHICS STATEMENT

This study was carried out in accordance with the ethical principles and guidelines set forth in the European Council Directive (86/609/EC) of 24 November, 1986, which outlines the protection of animals used for experimental purposes. The experiment was conducted under the approval of the state committee on animal ethics at Shiraz University, Shiraz, Iran (IACUC no: 4687/63).

### PEER REVIEW

The peer review history for this article is available at https://www.webofscience.com/api/gateway/wos/peer-review/10.1002/vms3.70024.

## Data Availability

Data available on request from the authors.
